# The prescription patterns of traditional Chinese medicine for women with polycystic ovary syndrome in Taiwan: A nationwide population-based study

**DOI:** 10.1097/MD.0000000000015890

**Published:** 2019-06-14

**Authors:** Mei-Jiun Lin, Hsiao-Wei Chen, Pi-Hua Liu, Wei-Jen Cheng, Shun-Li Kuo, Ming-Chen Kao

**Affiliations:** a Department of Traditional Chinese Medicine, Center for Traditional Chinese Medicine, Chang Gung Memorial Hospital; b School of Traditional Chinese Medicine; c Clinical Informatics and Medical Statistics Research Center, College of Medicine, Chang Gung University; d Department of Internal Medicine, Division of Endocrinology and Metabolism, Chang Gung Memorial Hospital, Taoyuan, Taiwan.

**Keywords:** complementary and alternative medicine, longitudinal health insurance database, polycystic ovary syndrome, traditional Chinese medicine

## Abstract

Polycystic ovary syndrome (PCOS) is a common endocrine disease of reproductive-age women, accounting for about 9% to 18% of all women in this age group. Hyperandrogenemia, oligomenorrhea, or amenorrhea or anovulation, and polycystic ovary morphology are the 3 main criteria used to diagnose PCOS currently. Substantial scientific evidence and consensus on treating Taiwanese PCOS was lacking. The aim of this study is to investigate the characteristics and utilization of traditional Chinese medicine (TCM) among Taiwanese women with PCOS.

The data used in this study were derived from the Longitudinal Health Insurance Database (LHID 2000 and LHID 2005). Demographic characteristics, TCM usage, the frequency, as well as average daily dose of Chinese herbal formulas and the single herbs prescribed for patients with PCOS, were analyzed. Chinese herbal formulas and the single herbs prescribed for PCOS women during 1999 to 2013 were extracted to build up Chinese Herbal Medicine prescription database.

In our study, 66.43% (n = 8205) women sought TCM treatment because of PCOS for infertility or menstrual disorders. The most commonly prescribed Chinese herbal formula was Jia-wei-xiao-yao-san (Supplemented Free Wanderer Powder). The most commonly prescribed single herb was Yi-mu-cao (*Leonuri herba*). Among top 20 Chinese herbal formulas, Si-wu-tang has the largest average daily dosage (9.60 g).

Our study identified the characteristics and prescription patterns of TCM for patients with PCOS in Taiwan. We may need do further longitudinal research for TCM and its long-term response for improvement of pregnancy rate and reduction of metabolic disease rate.

## 1 Introduction

Polycystic ovary syndrome (PCOS) is a common endocrine disease of reproductive-age women, accounting for about 9% to 18% of all women in this age group.^[1]^ Hyperandrogenemia, oligomenorrhea or amenorrhea or anovulation, and polycystic ovary morphology are the 3 main criteria used to diagnose PCOS currently. Clinical presentations of women with PCOS vary in different phenotypes, ages, ethnicities, and body weights. Obesity is a prominent feature of PCOS, occurring in 40% to 50% of these patients.^[2]^ However, the average body mass index was much lower in Taiwanese PCOS women than Western women.^[2]^ It is also important to evaluate the differences of PCOS women of various ages because of the clinical features and metabolic complications with the change of age.^[3,4]^

The treatment of PCOS is still challenging for physicians. The treatments on PCOS patients are mainly adopted depending on the symptoms.^[5]^ PCOS treatment depends primarily on the desired clinical effect including infertility treatment, regulation of menstrual disturbances, alleviation of the symptoms of hyperandrogenism, or obesity treatment. For women wishing to conceive, clomiphene still remains first-line therapy.^[6]^ PCOS patients whose goal is not pregnancy are usually advised to use oral contraceptives, which could correct menstrual abnormalities and hyperandrogenemia.^[6,7]^ However, treatment with oral contraceptives would increase the risk of venous thromboembolism.^[8]^ Complementary and alternative therapies, such as traditional Chinese medicine (TCM), have beneficial effects of decreasing risk.

Previously, we have retrospectively collected and analyzed the patients with PCOS at the Taoyuan Chang Gung Memorial Hospital between 2004 and 2013, and the results demonstrated that tonifying recipes were the most common prescribed herbal formula.^[9]^ Blood-regulating recipes and reconciliatory recipes were also commonly prescribed.^[9]^ The limitations of that study were small size of database and that most patients came from North Taiwan. Substantial scientific evidence and consensus on treating Taiwanese PCOS was lacking. The aim of this study is to investigate the characteristics and utilization of TCM among Taiwanese women with PCOS. Therefore, we could design clinical trials to draw a conclusion of the therapeutic effects in the future.

## 2 Materials and Methods

### 2.1 Data source and study subjects

The data used in this study were derived from the Longitudinal Health Insurance Database (LHID 2000 and LHID 2005). The database consists of a random sample of 1 million subjects selected from the 22 million insured people of the National Health Insurance (NHI) Program in 2000 and 2005, respectively. The program covers approximately 99% of the entire population of Taiwan because the program is a single-payer system with mandatory enrollment.^[10]^ The LHID contained all registration files and original claim data for reimbursement, as well as patient identification numbers, sociodemographic factors, diagnoses, prescription drugs dispensed, medical cost, medical care facilities, and specialties. The Ethics Committee of the Chang Gung Memorial Hospital waived the need for review board approval.

**Figure 1:**
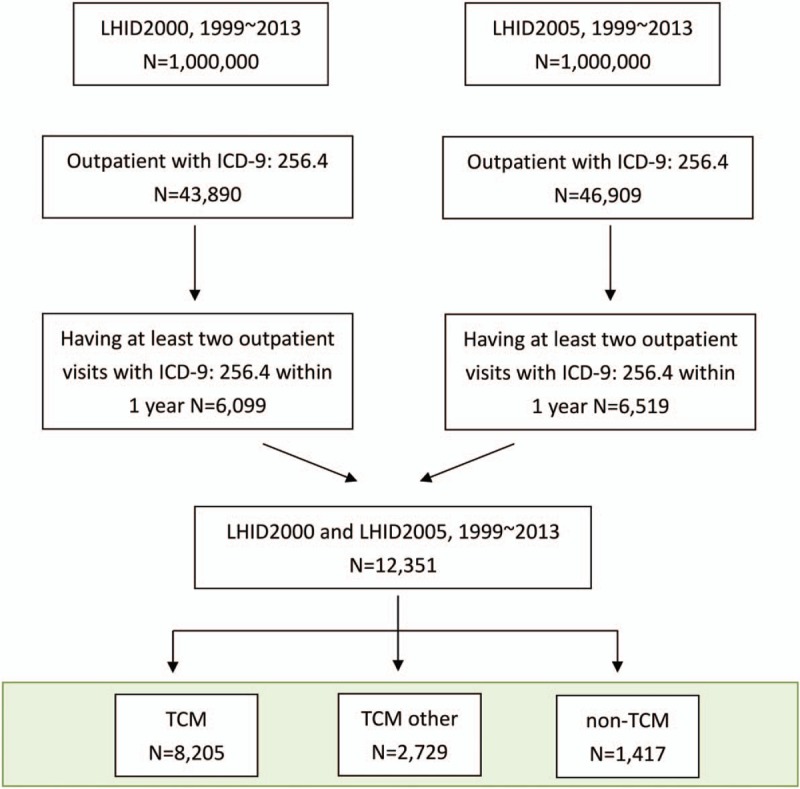
Polycystic ovary syndrome patient enrollment.

We included 12,351 individuals who had at least 2 clinic visits with diagnosis code of PCOS (*International Classification of Diseases, 9th Revision, Clinical Modification [ICD-9] codes: 256.4*) within 1 year from 1999 to 2013. All subjects were further divided into 2 groups: all TCM users (N = 10,934) and non-TCM users (N = 1417). To classify the treatment purpose, we specify all TCM users as 2 subgroups. One is TCM group (N = 8205), defined as patients with diagnosis of PCOS while them visited TCM clinics due to menstrual disorder or infertility problem. The other is “TCM other” group (N = 2729), defined as patients with diagnosis of PCOS and had least once visited TCM after the initial diagnosis of PCOS, but not for menstrual disorder or infertility problem. Non-TCM users (N = 1417) were defined as patients who never visited TCM clinics after the initial diagnosis (Fig. 1).

### 2.2 Statistical methods

Categorical data were presented as frequencies and percentages, and continuous variables were expressed as mean and standard deviation. Descriptive statistics were used to illustrate demographics and major disease categories in TCM and non-TCM users. *χ*^2^ and *t* tests were used to evaluate differences in the distribution of demographic, clinical characteristics, and major disease categories between the TCM and Non-TCM groups. A *P* value of <0.05 was considered statistically significant. All analyses were performed using SAS version 9.4 (SAS Institute Inc. Cary, NC).

## 3 Results

According to the age distribution, younger patients were more likely to seek TCM treatment. Most of the patients went to regional hospitals. Because Taiwan's population is mostly distributed in the North, so the patients are mostly in the North, followed by South Taiwan. Furthermore, TCM users had more outpatient visits per year than non-TCM users (*P* < .0001). Details on demographic distribution of TCM users and non-users are provided in Table 1.

**Table 1:**
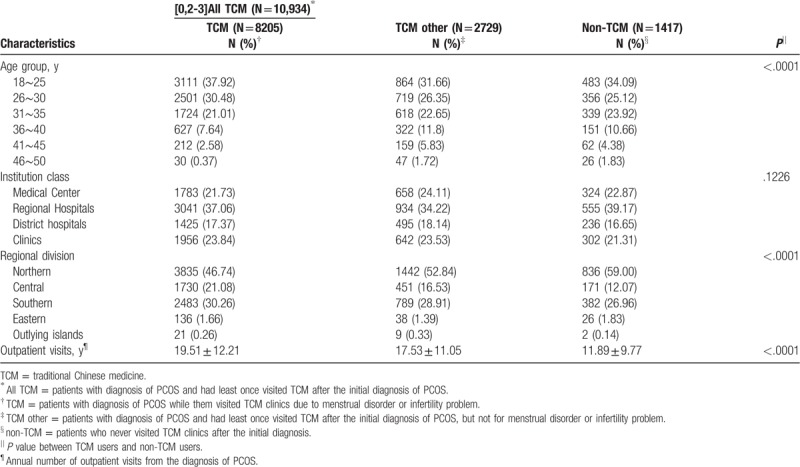
Demographic and clinical characteristics of TCM and non-TCM uses in patients with polycystic ovarian syndrome.

In TCM, there was no “PCOS” medical terminology, but with clinical symptoms such as amenorrhea, oligomenorrhea, irregular menstruation, or infertility. Therefore, we use Western medical diagnostic methods (*ICD-9*) for disease classification. We analyzed the distribution of major disease categories/diagnosis in TCM and non-TCM users among polycystic ovarian syndrome patients, which showed the major disease category for PCOS patients were endocrine, nutritional, and metabolic disease, followed by disease of genitourinary system. (Table 2)

**Table 2:**
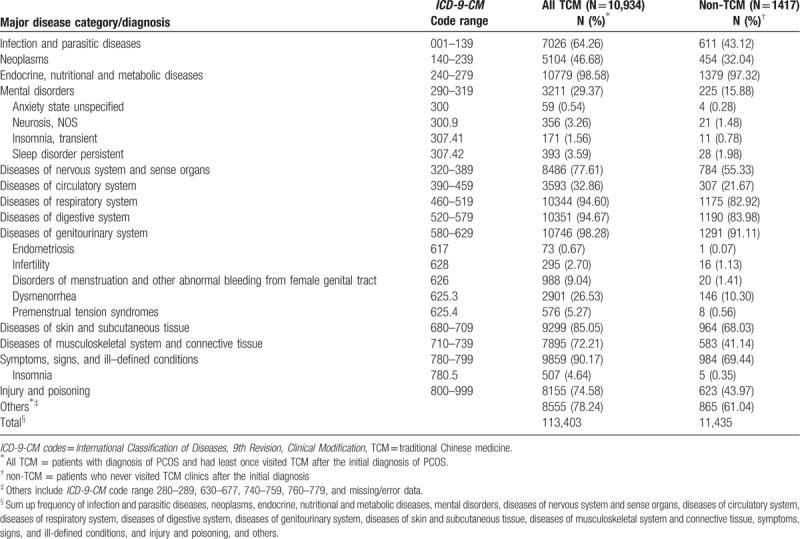
Distribution of major disease categories/diagnosis in TCM and non-TCM users among polycystic ovarian syndrome patients.

In Taiwan, there are 2 types of Chinese herbal medicine (CHM): herbal formulas and single herbs. Herbal formulas are mixtures of several herbal medicines and had specific indications for TCM use. Both herbal formulas and single herbs are all processed into concentrated powders. To investigate the prescription patterns of the Chinese herbal products for PCOS patients with infertility or menstrual disorders, we conducted a comprehensive analysis and identified 20 most commonly prescribed Chinese herbal formula (Table 3) and 10 single herbs (Table 4). The top 20 frequently used herbal formulas with its constituents and indication in TCM use are showed in Table 5 . The most commonly prescribed Chinese herbal formula was Jia-wei-xiao-yao-san (Supplemented Free Wanderer Powder), followed by Wen-jing-tang (Mensens-Warming Decoction) and Gui-zhi-fu-ling-wan (Cinnamon Twig and Poria Pill). The most commonly prescribed single herb was Yi-mu-cao (*Leonuri Herba*), followed by Xiang-fu (*Cyperi Rhizoma*) and Tu-si-zi (*Cuscutae Semen*). Among top 20 Chinese herbal formulas, Si-wu-tang has the largest average daily dosage (9.60 g).

**Table 3:**
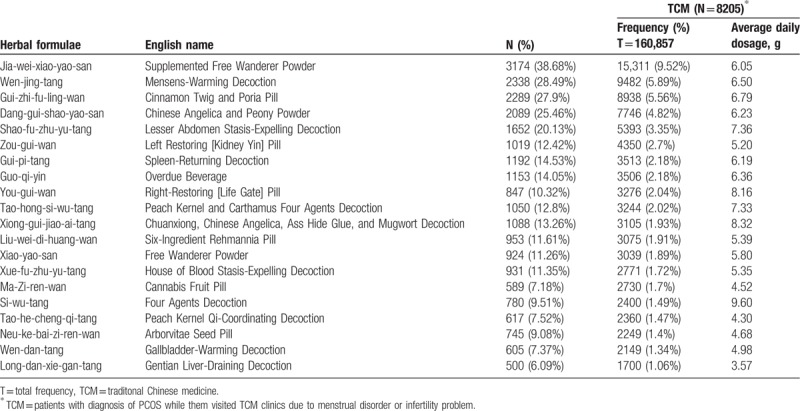
The top 20 most herbal formulas for patients with polycystic ovarian syndrome from1999 to 2013 in Taiwan.

**Table 4:**
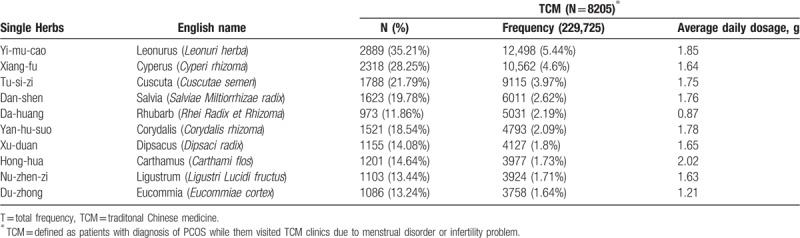
The most common prescribed single herbs in the treatment of polycystic ovarian syndrome.

**Table 5:**
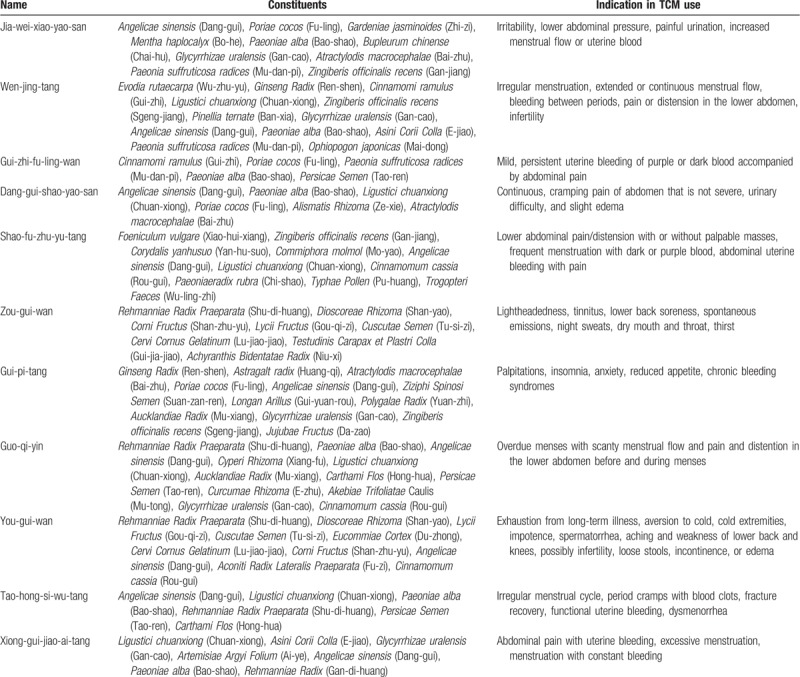
The top 20 most frequently used herbal formula with its constituents and indication in TCM use for polycystic ovary syndrome.

**Table 5 (Continued):**
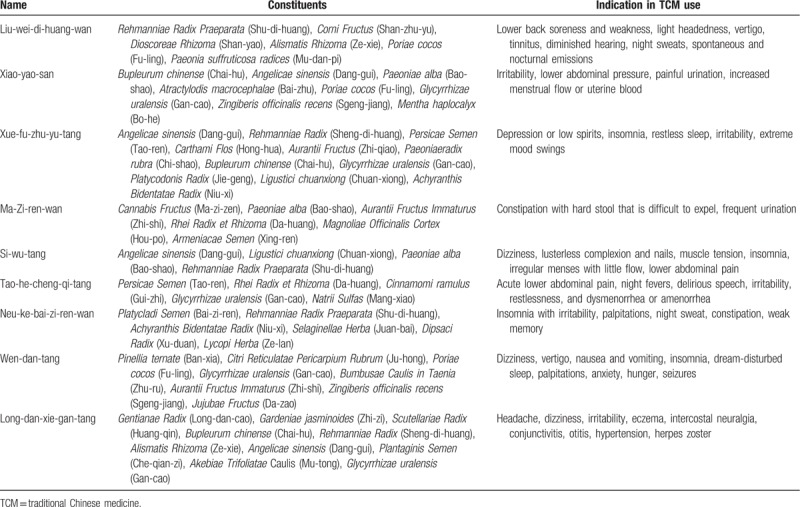
The top 20 most frequently used herbal formula with its constituents and indication in TCM use for polycystic ovary syndrome.

**Figure 2:**
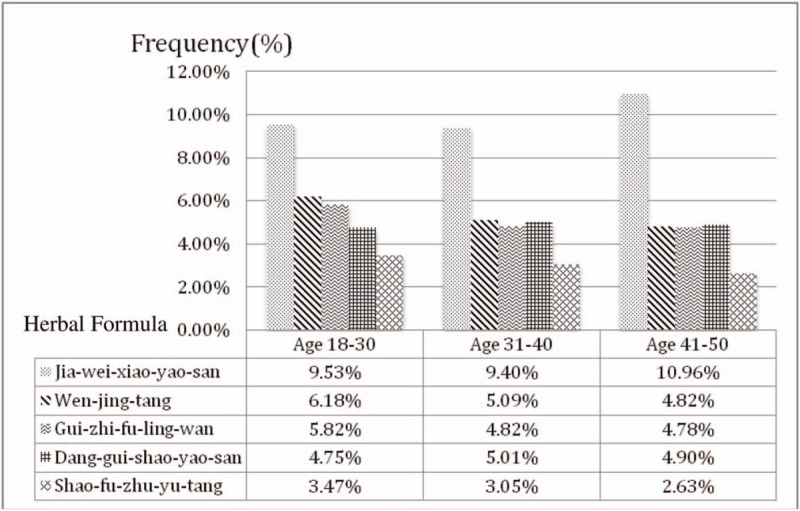
The top 5 most prescribed drugs for polycystic ovary syndrome startified by age group.

Owing to the clinical features and metabolic complications of PCOS with change of age,^[11,12]^ we grouped these patients to analyze their medication.^[9]^ The results showed Jia-wei-xiao-yao-san, Wen-jing-tang, Gui-zhi-fu-ling-wan, Dang-gui-shao-yao-san, and Shao-fu-zhu-yu-tang were the most 5 commonly prescribed Chinese herbal formulas at different age groups (Fig. 2). But the prescription pattern had no significant difference between different age groups.

## 4 Discussion

PCOS is a heterogeneous and complex disorder that has both adverse reproductive and metabolic implications for affected women.^[13]^ Many western medical therapies have been used to manage PCOS, such as oral contraceptives, insulin sensitizers, and laparoscopic ovarian drilling. CHMs have been suggested as an alternative approach for women with PCOS.^[14]^ This is a large-scale survey of Chinese herbal prescriptions and herbs used in the treatment of women with PCOS.

In Taiwan, the NHI provides coverage for >98% of the population, and the prescription for CHM and western medicine is equally covered. Therefore, patients are free to choose different treatments. According to Table 1, we found younger patients were more likely to seek for TCM treatment. In addition to the treatment of western medicine, TCM offers another treatment option, which may have fewer side effects.

In TCM, the major therapeutic principles of PCOS include tonifying the kidney, dispersing stagnated liver Qi, regulating blood, and clearing damp and resolving phlegm.^[9]^ Because every prescription has their major effects matching the therapeutic principles, we analyze the prescriptions and classify their major effects into different categories. The most common category was dispersing stagnated liver Qi, which may be caused by modern social or family pressures. The common category was followed by dispersing stagnated liver Qi, tonifying the kidney/qi/blood and regulating blood. Therefore, in Table 3, the top 6 drugs are Jia-wei-xiao-yao-san, Wen-jing-tang, Gui-zhi-fu-ling-wan, Ang-gui-shao-yao-san, Shao-fu-zhu-yu-tang, and Zou-gui-wan.^[9]^

The most commonly prescribed herbal formula, Jia-Wei- Xiao-Yao-San, has been used for thousands of years to treat women's disorder ranging from menstrual problems, infertility to menopausal syndromes.^[15]^ It had been demonstrated that this prescription could be used for women who are prone to mood disorders and anxiety.^[16]^ Jia-Wei-Xiao-Yao-San had also been found to increase levels of tumor necrosis factor-α,^[17]^ which might affect mood and emotional status. Wen-jing-tang, Gui-zhi-fu-ling-wan, and Dang-gui-shao-yao-san are conditioning menstruation and regulating blood, which is also common in PCOS patient. Guo-qi-yin, Tao-hong-si-wu-tang, Si-wu-tang, Xue-fu-zhu-yu-tan, and Neu-ke-bai-zi-ren-wan also could promote blood circulation and remove blood stasis. Si-wu-tang could decrease serum follicle-stimulating hormone/luteinizing hormone ratio and testosterone level in PCOS patients. Besides, Si-wu-tang may have the effects of promoting follicle development and establishing regular menstruation cycles.^[18]^ Qui-pi-tan could supplement qi and blood, which can improve the menopausal symptoms and increased the locomotor activity, thereby increasing bone mineral density in ovariectomized rat model.^[19]^ Wen-jing-tang and Dang-gui-shao-yao-san are able to correct luteal insufficiency and Gui-zhi-fu-ling-wan has anti-estrogen effect.^[20]^ Zou-gui-wan, You-gui-wan, and Liu-wei-di-huang-wan are tonifying the kidney. Zou-gui-wan and You-gui-wan treating human reproductive dysfunctions may be through an enhancement of neooogenesis.^[21]^ Liu-wei-di-huang-wan could significantly reduce the levels of follicle-stimulating hormone and luteinizing hormone and increase the level of estrodial.^[22]^

In older women, metabolic disturbances and obesity are the major problem.^[11]^ We usually prescribe Wen-dan-tang to clear damp and resolve phlegm from obese patients in TCM classics. However, in Table 3, Wen-dan-tan is not one of the top categories in this study. One reason could be that obese female population is relatively small in Taiwan. In addition to this, in the top 20 commonly used drugs, Long-dan-xie-gan-tang belongs to clearing away heat purge pathogenic fire category, which is not within the 4 major therapeutic principles, but still common for PCOS patients. Besides, in other animal experiment, Long-dan-xie-gan-tang could increase corpora lutea and corpora albicantia, whereas cystic follicles and secondary follicles numbers were decreased.^[23]^

Table 4 shows top 10 most common prescribed single herbs and classifies their major effects into different categories. Most of them belong to the category of promoting blood circulation and removing blood stasis, such as Yi-mu-cao, Dan-shen, Yan-hu-suo, and Hong-hua. The others belong to tonifying the kidney, such as Tu-si-zi, Xu-duan, Nu-zhen-zi, and Du-zhong. The top first drug is Yi-mu-cao, which was reported to have anti-oxidative activity and could treat dysmenorrhea by relaxing uterine spasms, decreasing inflammation, reducing prostagaldin F2α, and prostaglandin synthase-2 concentration in uterine smooth muscle and increasing the serum progesterone level.^[24,25]^ Xiang-fu, the second commonly used drugs, also has antioxidative potency and free radical scavenging activity.^[26]^ The third common prescribed drug is Tu-si-zi, which improves ovarian endocrine dysfunction and increases estrogen receptor expression in the hippocampus, hypothalamus, and pituitary glands, as well as luteinizing hormone receptor expression in the ovaries.^[27]^ What is worth noting is Hong-hua, which has the largest average daily dose. According to research, Hong-hua is effective in relieving symptoms of premenstrual syndrome and treating primary dysmenorrhea.^[28,29]^

According to previous research, hyperandrogenism and chronic anovulation were the primary disturbances in younger women with PCOS, whereas,obesity, insulin resistance, and metabolic disturbances were predominant in older women with PCOS.^[11,12]^ For hyperandrogenism, the common symptoms were hirsutism and acne, which we usually prescribed Chinese medication of clearing away heat purge pathogenic fire category for treatment. But for these symptoms, we use single herb relatively often in prescriptions. For the symptom of chronic anovulation, we usually prescribe categories of tonifying the kidney, dispersing stagnated liver Qi, and regulating blood. Figure 2 shows the most common prescribed Chinese herbal formula in different age groups. However, there are no significant differences between these groups. The result may indicate that regardless of the age of the treatment guidelines are the same.

Besides, we reviewed the pattern about CHM for PCOS treatment in China. The result showed different trials chose different medications based on their clinical experiences. But the treatment pattern was similar to our study: tonifying the kidney, dispersing stagnated liver Qi, and regulating blood.^[14]^ Different TCM doctors may not prescribe the same herbal formula, but we used the same principle to treat.

After this research, we may understand the CHM treatment patterns of Taiwanese PCOS patients. However, there are several limitations in this study. First, except CHM treatment, there are other treatment ways for PCOS treatment, like acupuncture or folk medicine. We did not analyze acupuncture treatment in this study because we only wanted to see CHM treatment status. The NHI did not reimburse the folk medicine in Taiwan. Therefore, folk medicine was excluded in this study. Second, the therapeutic effectiveness is unknown, such as regular menstruation, presence or absence of ovulation, improvement of pregnancy rate, and reduction of metabolic disease rate. Third, the compliance of taking prescription was not revealed in the database. So, we do not know whether the patients actually took the CHM. Further well-designed clinical trials are necessary on the basis of these results to clarify the efficacy of CHM.

## 5 Conclusion

This is the first study about large-scale pharmacoepidemiological analysis on TCM prescriptions for PCOS. The study illustrated the top herbal formulas and single herbs which are within these categories: dispersing stagnated liver Qi, conditioning menstruation, tonifying the kidney/qi/blood, and regulating blood. These results are also the same as PCOS treatment principles in TCM. Based on Fig. 2, different age group PCOS patients prescribed similar herbal formula. Therefore, it could be used as a reference for clinical prescription. Further well-designed clinical trials are needed to draw a conclusion of the therapeutic effects.

## Author contributions

**Conceptualization:** Lin Mei-Jiun, Kao Ming-Chen.

**Data curation:** Liu Pi-Hua

**Formal analysis**: Liu Pi-Hua, Lin Mei-Jiun, Chen Hsiao-Wei, Kao Ming-Chen

**Investigation**: Lin Mei-Jiun, Chen Hsiao-Wei, Kao Ming-Chen

**Methodology**: Liu Pi-Hua, Kao Ming-Chen

**Software**: Liu Pi-Hua, Kao Ming-Chen

**Supervision**: Cheng Wei-Jen, Kuo Shun-Li, Kao Ming-Chen

**Validation**: Cheng Wei-Jen, Kuo Shun-Li

**Writing – original draft**: Lin Mei-Jiun, Chen Hsiao-Wei

**Writing – review & editing**: Cheng Wei-Jen, Kuo Shun-Li, Kao Ming-Chen

**Formal analysis:** Pi Hua Liu.

**Methodology:** Wei Jen Cheng, Shun Li Kuo.

**Writing – original draft:** Mei Jiun Lin, Hsiao Wei Chen.

**Writing – review & editing:** Ming Chen Kao.
